# SENP1 Senses Oxidative Stress to Regulate the SUMOylation Modification of ZIP8 and Maintain Zinc Transport Functions

**DOI:** 10.3390/antiox14060750

**Published:** 2025-06-18

**Authors:** Tao Liu, Chang-Chun Song, Fu-Xuan Duan, Chong-Chao Zhong, Sheng-Zan Liu, Jia-Cheng Guo, An-Gen Yu, Zhi Luo

**Affiliations:** 1 Hubei Hongshan Laboratory, Fishery College, Huazhong Agriculture University, Wuhan 430070, China; taoliu@webmail.hzau.edu.cn (T.L.); songchangchun@webmail.hzau.edu.cn (C.-C.S.); duanfx@webmail.hzau.edu.cn (F.-X.D.); zhongchongchao@webmail.hzau.edu.cn (C.-C.Z.); lsz2020@webmail.hzau.edu.cn (S.-Z.L.); jiachengguo@webmail.hzau.edu.cn (J.-C.G.); yuangen1996@webmail.hzau.edu.cn (A.-G.Y.); 2 Laboratory for Marine Fisheries Science and Food Production Processes, Qingdao Marine Science and Technology Center, Qingdao 266237, China

**Keywords:** Zinc, Zinc transporters, SUMOylation modification, Zn homeostasis, oxidative stress

## Abstract

Zinc (Zn) is a crucial trace element in vertebrates, fulfilling a range of physiological functions, whose metabolism and homeostasis are manipulated by Zn transporter proteins. SUMOylation, a reversible post-translational modification (PTM), extensively participates in various biological processes in the body, yet its underlying mechanism in regulating Zn transporters remains unexplored. Our findings indicate that high dietary Zn substantially elevated intestinal Zn content and modulated the expression profiles of Zn transporter-related genes and proteins, including ZIP8 transporter. In addition, high Zn diet tended to inhibit the SUMOylation modification and upregulate deSUMOylation modification in the intestine and intestinal epithelial cells. Furthermore, we found that the ZIP8 protein undergoes SUMOylation modification; UBC9 upregulated but SENP1 and Zn downregulated the SUMOylation level of ZIP8, and the K24 and K222 positions are the primary SUMOylation modification sites of ZIP8 protein in yellow catfish. Mechanistically, SENP1 modulates the deSUMOylation modification of ZIP8 by sensing Zn-induced oxidative stress. In summary, for the first time, we have uncovered a unique regulatory mechanism of ZIP8 mediated by SUMOylation modification in vertebrates and demonstrate that SENP1 is capable of sensing oxidative stress to reduce the SUMOylation modification of ZIP8 at K24 and K222 sites.

## 1. Introduction

Zinc (Zn) plays a crucial role in organisms, involved in various physiological function including DNA synthesis, protein translation, growth, and immune responses in vertebrates [1,2]. Furthermore, Zn is a trace element second only to iron in the body and is essential for maintaining the normal functions of over 300 enzymes and more than 1000 transcription factors [3,4]. Zn deficiency can induce a range of diseases, such as growth retardation, impaired immune function, skin damage, and reproductive dysfunction [5,6]. Alternatively, excessive accumulation of Zn in the body contributes to cytotoxicity, which triggers adverse reactions such as oxidative stress and metabolic imbalance [7,8]. Zn also plays an important role in maintaining the integrity of the intestinal mucosal barrier. Therefore, it is extremely important to maintain Zn homeostasis within the body.

The balanced state of Zn is coordinately controlled by its absorption, export, distribution, and storage [5]. Dietary Zn is absorbed by intestinal epithelial cells. The gastrointestinal tract serves as the main site for the absorption and excretion of Zn [9], and many functional proteins mediate these processes and jointly maintain the homeostasis of Zn in the body, such as Zn transporter families (ZIP and ZnT), metallothioneins (MTs) and metal-responsive transcription factor 1 (MTF-1) [5,10,11]. Generally, ZIP proteins are responsible for transporting Zn into the cytosol from the extracellular environment or intracellular compartments, whereas ZnT proteins facilitate the movement of Zn out of the cytosol and into the extracellular space or intracellular compartments [5]. MTs are capable of binding with Zn ions, thereby reducing free Zn in the cytoplasm and maintaining Zn homeostasis within the cell [12,13]. To date, MTF-1 serves as the only cellular Zn-sensing transcription factor in vertebrates, modulating intracellular metal ion homeostasis via binding to metal response elements (MRE) of its target genes [14,15]. ZIP8, belonging to the ZIP family, is localized at the plasma membrane and serves as a key mediator in increasing the cytosolic Zn concentration [16,17]. Understanding the regulatory mechanisms governing ZIP8 is crucial, given that dysregulation of its expression and activity contributes to the pathogenesis and progression of chronic diseases. However, the post-transcriptional regulatory mechanisms of ZIP8 protein remain unclear. Therefore, investigation into the function and regulatory mechanisms of this protein is significant for maintaining intracellular Zn homeostasis, thereby preventing various metabolic abnormalities caused by Zn imbalance.

Posttranslational modifications (PTMs), such as methylation, acetylation, ubiquitination, and the SUMOylation of targeting lysine residues, are indispensable for multiple cellular processes [18]. Among these PTMs, SUMOylation modification also participates in various biological processes by influencing the transcriptional activity, stability, and subcellular localization of target proteins in reaction to environmental changes and cellular stresses [19,20]. SUMOylation refers to the process in which small ubiquitin-related modifiers (SUMOs), such as SUMO1, SUMO2, and SUMO3, are covalently conjugated to the lysine residues of target proteins with the involvement of activating (E1, SAE1/UBA2), conjugating (E2, Ubc9), and ligating (E3) enzymes [21]. SUMOylation, a reversible modification, can be reversed by seven isoforms of sentrin/SUMO-specific proteases, collectively known as SENPs [20]. Among these SENPs, SUMO-specific protease 1 (SENP1) has been identified as a pivotal enzyme regulating the SUMOylation process and is associated with multiple diseases [22]. SENP1 primarily catalyzes the dissociation of SUMO1 from its target proteins and influences multiple cellular processes, such as protein stability, DNA repair, transcriptional regulation, and cellular responses to stress [23,24]. Several investigations have shown a marked elevation in the expression levels of SENP1 under stressful conditions, particularly oxidative stress [25,26]. Thus, SENP1 is highly sensitive to redox status, closely associated with oxidative stress, and participates in various physiological and pathological processes. Studies suggested that Zn deficiency or excess triggers oxidative stress by increasing the generation of reactive oxygen species across different cell lines and animal models [1,14]. However, whether SENP1 senses oxidative stress to control Zn transporters and homeostasis remains unexplored.

The world is endowed with abundant fisheries resources, housing approximately 30,000 species of fish, making fish the largest group of vertebrates on the planet [27]. Despite significant evolutionary divergence from mammals, fish exhibit remarkable similarities in terms of metabolic pathways within the organism and the absorption and transport of mineral elements [28,29]. The yellow catfish *Pelteobagrus fulvidraco*, a widely cultivated freshwater economic fish species both domestically and internationally, has had its whole-genome information published internationally [30] and accordingly are considered an excellent model for studying Zn metabolism in fish [28,31]. Furthermore, our previous research has completed the bioinformatics prediction of some SUMOylation modification sites of Zn transporter proteins [32]. However, the function of Zn transporter proteins and regulatory mechanisms by SUMOylation modification have yet to be explored. Although earlier in 2011, Liu et al. [33] published a paper in *J. Biol. Chem.* and revealed that the key transcription factor MTF-1 (zinc sensor) can undergo SUMOylation modification; they did not investigate the SUMOylation modification of Zn transporters. Therefore, this study aims to analyze the interaction among dietary Zn levels, body Zn metabolism, and SUMOylation modification while delving into the underlying mechanism through which SUMOylation modulates Zn homeostasis. Our research unveils a novel regulatory mechanism where SENP1 senses oxidative stress to modulate the SUMOylation level of ZIP8 at K24 and K222 sites and maintain its Zn transport function. These findings identify oxidative stress as a critical metabolic regulator in Zn metabolism and homeostasis and suggest that inhibiting SENP1 activity represents a promising strategy for alleviating high Zn-induced Zn homeostatic disorder and oxidative stress.

## 2. Materials and Methods

### 2.1. Ethical Statement

This research involves animal experimentation protocols that strictly adhere to the Guidelines for Laboratory Animals Care and Use established by Huazhong Agricultural University (HZAU) and have been authorized by the HZAU Experimental Animal Ethics Committee (code: FISH-2021-1015, 15 October 2021).

### 2.2. Animal Feeding, Management, and Sample Collection

The formulation of the feeding, breeding, and management approach for yellow catfish in this experiment were described in our recent study [34]. Three experimental diets were prepared, with the supplementation of ZnSO_4_·7H_2_O at concentrations of 0 g/kg (low-zinc group, L-Zn, without additional zinc), 0.03 g/kg (medium-zinc group, M-Zn), and 0.45 g/kg (high-zinc group, H-Zn). The Zn content in the feed and intestinal tissues was measured using ICP-OES (Optima 8000DV, PerkinElmer, Waltham, MA, USA). The final Zn content of the three experimental feeds was found to be 12.10 (L-Zn), 18.69 (M-Zn), and 120.82 (H-Zn) mg/kg, respectively. According to the study by Luo et al. [35], M-Zn group was optimal in meeting dietary Zn requirement for yellow catfish. The feeding experiment continued for 10 weeks. The particular experimental procedures and sampling methods are presented in Supplementary Materials Text S1.

### 2.3. Cell Culture and Treatments

The primary intestinal epithelial cells (IECs) were isolated from the intestinal tract of yellow catfish and cultured according to the methods described in our recently published literature [36]. Furthermore, the HEK293T cell lines were employed to analyze protein–protein interactions, SUMOylation sites, and related functions. The detailed culture methods of IECs and HEK-293T cells are presented in Supplementary Materials Text S2. To investigate whether oxidative stress mediates the changes in SENP1 protein expression induced by different Zn levels, the cells were treated with Zn (100 μM ZnSO_4_) or Zn chelator TPEN (N,N,N′,N′-tetrakis (2-pyridinylmethyl) ethylenediamine, 2 μM). To explore whether oxidative stress is involved in the regulation of SENP1 protein expression, IECs were treated with the ROS inducer H_2_O_2_ (hydrogen peroxide, 200 μM). To investigate whether oxidative stress mediates the changes in SENP1 protein expression induced by different Zn concentrations, IECs were treated with an ROS scavenger NAC (N-acetylcysteine, 0.5 mM). The cells were collected for subsequent analysis after being treated for 48 h.

### 2.4. Determination of Antioxidant Enzyme Activity and MDA Content

The activity of glutathione peroxidase (GPx, #S0058) in the intestinal tissues of yellow catfish was determined using a reagent kit from Beyotime Biotechnology (Shanghai, China). The catalase activity (CAT, #A007-1-1), total antioxidant capacity (T-AOC, #A015-1), and malondialdehyde (MDA, #A003-1) content were measured using commercial kits supplied by Nanjing Jiancheng Bioengineering Institute Co., Ltd. (Nanjing, China).

### 2.5. Reverse Transcription and Real-Time Quantitative PCR (qRT-PCR) Analysis

Total RNA was isolated from intestinal tissue using TRIzol reagent (Thermo Fisher Scientific, Waltham, MA, USA) in accordance with the manufacturer’s instructions. qPCR experiments were performed based on our previously published protocol [37]. The detailed methodology is outlined in Supplementary Materials Text S3. The 2^−ΔΔCt^ method was employed to quantify the mRNA expression levels. The specific primers used for RT-PCR are detailed in Supplementary Materials Table S1.

### 2.6. Western Blot Analysis, Immunoprecipitation, and Co-Immunoprecipitation Analysis

In this study, we analyzed the protein expression levels using Western blot, following the methods described in our previous research [37]. The detailed experimental procedures have been provided in Supplementary Materials Text S4. Quantitative analysis of the Western blot results was performed using Image-Pro Plus 6.0 software (Media Cybernetics, MD, USA).

Furthermore, immunoprecipitation and co-immunoprecipitation experiments were conducted to assess the SUMOylation status of ZIP8 and explore potential protein–protein interactions. These experimental were performed in accordance with our previous study [37], and detailed experimental steps are presented in Supplementary Materials Text S5.

### 2.7. Plasmid Construction and Transfection

To validate whether ZIP8 undergoes SUMOylation and to explore the SUMOylation sites and related functions of ZIP8, we constructed overexpression vectors for ZIP8, SUMO1, SUMO2, SUMO3, UBC9, and SENP1, referring to the established methods from previous research [37]. The detailed procedures are outlined in Supplementary Materials Text S6. The PCR primers used for gene cloning and site-directed mutagenesis are listed in Supplementary Materials Table S2. The methodology for cell transfection experiments was based on our recent study [37]. After 24 h, the cells were harvested for Western blot, immunoprecipitation, and co-immunoprecipitation analyses.

### 2.8. Statistical Analysis

All statistical analyses in this study were performed using SPSS 19.0 software (Armonk, NY, USA). All data were presented as mean ± SEM (standard error of the means) from a minimum of three independent experiments. Differences among the three treatments groups (low Zn, middle Zn, and high Zn) were evaluated using one-way analysis of variance (ANOVA) followed by a post hoc Duncan’s multiple range test. For comparisons between two groups, Student’s *t*-test was applied. A *p*-value below 0.05 was considered statistically significant.

## 3. Results

### 3.1. In Vivo Studies

#### 3.1.1. High Zn Diet Increased Zn Accumulation and Induced Oxidative Stress of Intestinal Tissues

**Figure 1:**
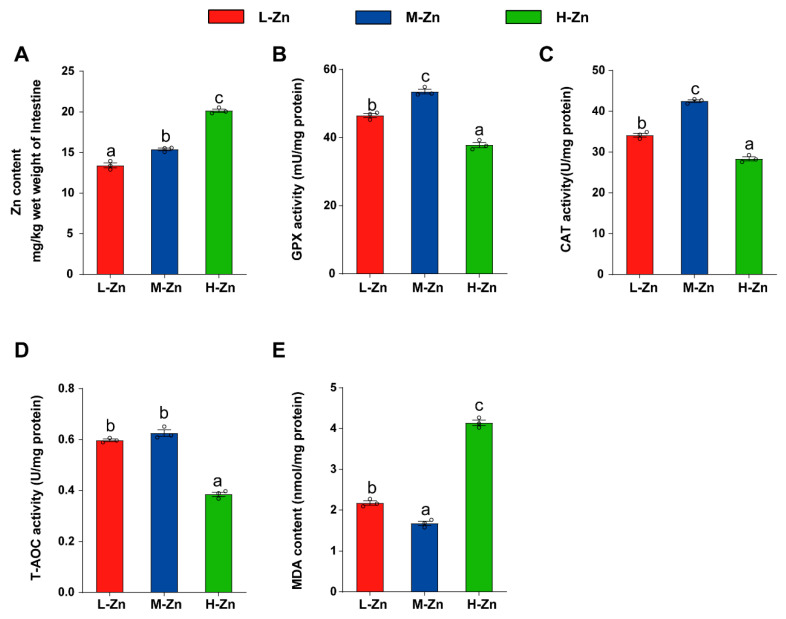
The impact of dietary Zn levels on Zn content, antioxidant enzyme activities, and MDA content in the intestine of yellow catfish. (**A**) Zn content. (**B**) GPX activity. (**C**) CAT activity. (**D**) T-AOC activity. (**E**) MDA content. Values are means ± SEM (*n* = 3 replicate tanks, 6 fish per tank). Values with different letters (a–c) within the same chart are significantly different at *p* < 0.05.

The intestinal Zn content gradually increased with the addition of dietary Zn (*p* < 0.05) (Figure 1A). Among the three groups, the H-Zn group possessed the lowest activities of GPx, CAT, and T-AOC, while the M-Zn group showed the highest (*p* < 0.05) (Figure 1B–D). Moreover, the H-Zn group exhibited the highest malondialdehyde (MDA) content, while the M-Zn group showed the lowest (*p* < 0.05) (Figure 1E). The above results demonstrated that a high dietary Zn elevated Zn content and induced oxidative stress in the intestine.

#### 3.1.2. Dietary Zn Supplementation Affects Intestinal Zn Absorption and Transport

**Figure 2:**
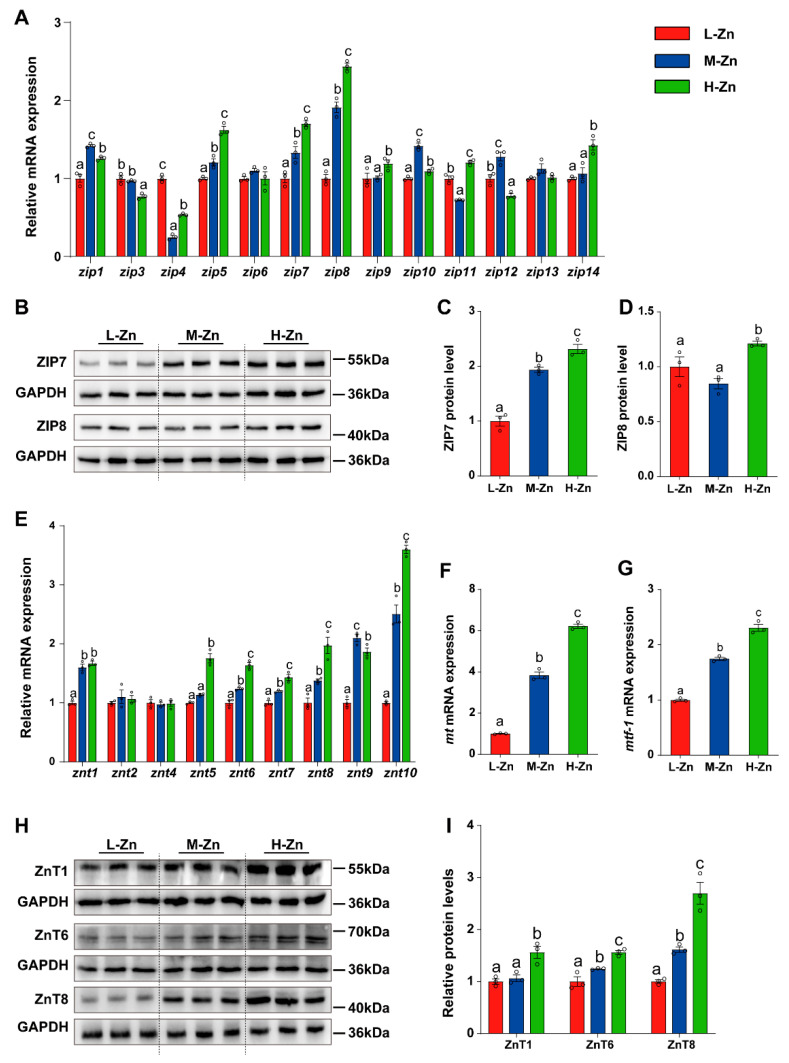
The effect of dietary Zn levels on the expression of genes related to Zn absorption and transport in the intestine of yellow catfish. (**A**) qPCR analysis of mRNA expression of SLC39A (ZIPs) family genes. (**B**–**D**) Western blot (**B**) and quantitative analysis of ZIP7 (**C**) and ZIP8 (**D**) protein expression. (**E**–**G**) qPCR detection of the mRNA expression of SLC30A (ZnTs) family (**E**), *mt* (**F**), and *mtf*-1 (**G**). (**H**,**I**) Western blot and quantitative analysis of ZnT1, ZnT6, and ZnT8 protein expression. Values are means ± SEM (*n* = 3 replicate tanks, 6 fish per tank). Values with different letters (a–c) within the same chart are significantly different at *p* < 0.05.

To further explore the influence of dietary Zn supplementation on intestinal Zn absorption and transport, the mRNA and protein expression of the SLC39A (ZIPs) family, SLC30A (ZnTs) family, MT, and MTF-1 was measured in the intestine (Figure 2). In general, dietary Zn levels differentially influenced expression of ZIPs and ZnTs family, MT, and MTF-1 (Figure 2). For the SLC39A (ZIPs) family, the mRNA levels of *zip5*, *zip7*, and *zip8* gradually increased with the addition of dietary Zn. In comparison to the other two groups, H-Zn increased *zip9* and *zip14* mRNA expression, but downregulated the *zip3* mRNA level. Among three dietary groups, fish fed the M-Zn diet showed the highest mRNA levels for *zip1*, *zip10*, and *zip12* and the lowest for *zip4* and *zip11*. Dietary Zn supplementation showed no impact on the mRNA abundance of *zip6* and *zip13* (*p* < 0.05) (Figure 2A). Furthermore, the protein levels of ZIP7 and ZIP8 gradually increased with the addition of dietary Zn (*p* < 0.05) (Figure 2B–D).

For SLC30A (ZnTs) family, the mRNA levels of *znt6*, *znt7*, *znt8*, and *znt10* were upregulated with an increase in dietary Zn levels. In comparison to the other two groups, L-Zn diet significantly downregulated the *znt1* mRNA expression. Compared with the L-Zn and M-Zn group, H-Zn diet markedly increased the mRNA abundance of *znt5*. Among the three dietary groups, the M-Zn group exhibited the highest mRNA abundance of *znt9*, whereas the L-Zn group showed the lowest. Moreover, there was no significant impact of dietary Zn supplementation on the mRNA abundances of *znt2* and *znt4* (*p* < 0.05) (Figure 2E). Furthermore, an elevation in dietary Zn levels was accompanied by a significant increase in *mt* and *mtf-1* mRNA expression (*p* < 0.05) (Figure 2F,G). Compared with the L-Zn and M-Zn group, high Zn diet also increased the protein expression of ZnT1(*p* < 0.05) (Figure 2H,I). Moreover, the protein levels of ZnT6 and ZnT8 augmented in conjunction with the elevation of dietary Zn levels (*p* < 0.05) (Figure 2H). Consequently, our results indicated that high Zn diet increased intestinal Zn intake, thereby affecting the expression of genes related to Zn absorption and transport in the intestine.

#### 3.1.3. Dietary Zn Levels Affected the Expression of Genes and Proteins Associated with SUMOylation Modification

**Figure 3:**
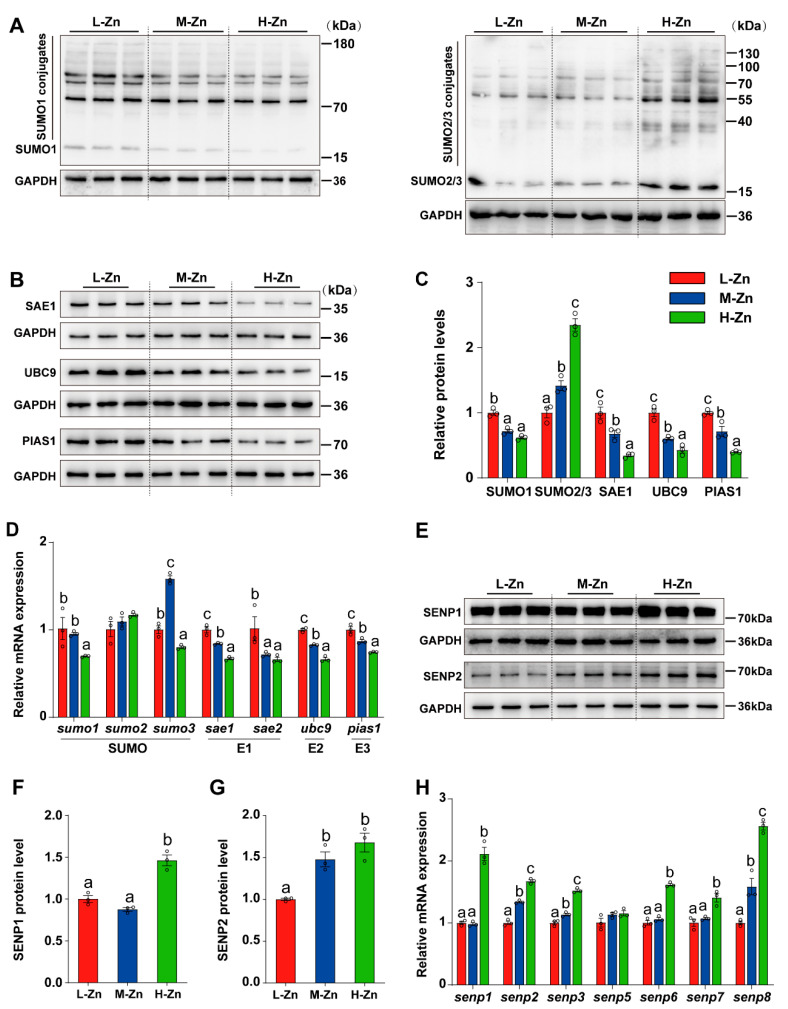
The impact of dietary Zinc levels on the expression of SUMOylation-related genes and proteins in the intestine of yellow catfish. (**A**–**C**) Western blot and quantitative analysis of SUMO1, SUMO2/3, SAE1, UBC9, and PIAS1. (**D**) qPCR analysis of intestinal SUMOylation-related genes. (**E**–**G**) Western blot and quantitative analysis of SENP1 (**E**,**F**) and SENP2 (**E**,**G**). (**H**) qPCR detection of intestinal deSUMOylation-related genes. Values are means ± SEM (*n* = 3 replicate tanks, 6 fish per tank). Values with different letters (a–c) within the same chart are significantly different at *p* < 0.05.

Since Zn can regulate SUMOylation modification [38], the impact of dietary Zn levels on the expression of genes related to SUMOylation at the mRNA and protein levels was investigated. The protein expression of SUMO1 was markedly elevated in L-Zn group compared to the other two groups (*p* < 0.05) (Figure 3A,C). With the increasing dietary Zn levels, the protein expression of SAE1, UBC9, and PIAS1 were decreased (*p* < 0.05) (Figure 3B,C), while the protein levels of SUMO2/3 was increased (*p* < 0.05) (Figure 3A,C). Moreover, in comparison to the L-Zn and M-Zn groups, a high dietary Zn resulted in a marked decline in the mRNA expression of *sumo1*. The mRNA abundance of *sumo3* was the highest in the M-Zn group and lowest in the H-Zn group. In addition, we also found that the mRNA abundances of *sae1*, *ubc9*, and *pias1* declined with dietary Zn addition. Fish fed with L-Zn diet had a significantly higher mRNA abundance of *sae2* compared to those in the M-Zn and H-Zn groups (*p* < 0.05) (Figure 3D). Furthermore, dietary Zn supplementation did not exert any impact on *sumo2* mRNA expression (Figure 3D).

In addition, the mRNA abundance of genes related deSUMOylation was also determined. Our study found that high Zn diet supplementation increased the protein level of SENP1 compared to the L-Zn and M-Zn groups (*p* < 0.05) (Figure 2E,F). The SENP2 protein levels in the H-Zn group were higher compared to the L-Zn group but showed no difference from the M-Zn group (*p* < 0.05) (Figure 2E,G). Moreover, our analysis revealed an upregulation in the mRNA abundances of *senp2*, *senp3*, and *senp8* in response to increased dietary Zn levels (*p* < 0.05) (Figure 2H). In comparison with the L-Zn and M-Zn groups, high Zn diet also increased the mRNA abundances of *senp1*, *senp6*, and *senp7* (*p* < 0.05) (Figure 2H). Dietary Zn supplementation did not alter the mRNA expression of *senp5* (Figure 2H).

### 3.2. In Vitro Studies

#### 3.2.1. The ZIP8 Protein Undergoes SUMOylation Modification

**Figure 4:**
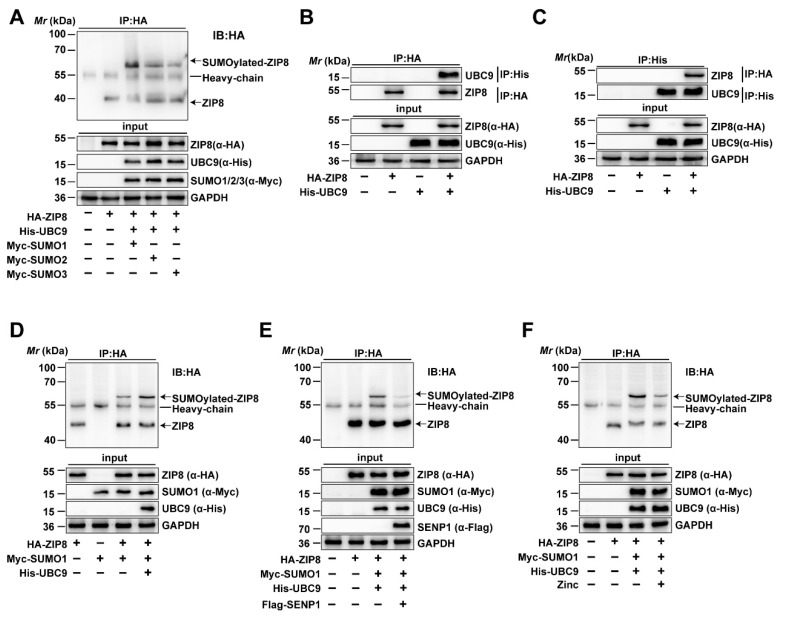
ZIP8 can undergo SUMOylation modification. (**A**) ZIP8 is SUMOylated by SUMO1, SUMO2, and SUMO3. HEK-293T cells were transfected with HA-ZIP8 and Myc-tagged SUMO1, SUMO2, and SUMO3. Subsequently, cell lysates were subjected to IP using anti-HA antibodies and then immunoblotted with the same antibodies. The SUMOylated bands of ZIP8 were marked with arrows. (**B**,**C**) The interaction between ZIP and UBC9. HEK-293T cells overexpressing HA-ZIP8 and His-UBC9 were lysed and then subjected to IP treatment using anti-HA (**B**) or anti-His (**C**) antibodies, immunoblotting analysis was then performed using corresponding antibodies. (**D**) UBC9 Enhances SUMOylation of ZIP8. The co-transfection of Myc-SUMO1 and HA-ZIP8 into HEK-293T cells, with or without the transfection of His-UBC9, was performed. The SUMOylation of ZIP8 was Identified using anti-Myc or anti-HA antibodies. The SUMOylated bands of ZIP8 are marked with arrows. (**E**) SENP1 deSUMOylated ZIP8. HEK-293T cells were transfected with HA-ZIP8 and Myc-SUMO1, and the SUMOylation of ZIP8 was detected using specific antibodies. (**F**) Zn incubation inhibits the SUMOylation of ZIP8. HA-ZIP8, Myc-SUMO1, and His-UBC9 were transfected into HEK-293T cells and incubated with or without 100 μM Zn for 24 h. IP was performed using anti-HA antibodies. Subsequently, immunoblotting was conducted using the identical antibody to determine the SUMOylation state of ZIP8 when exposed to Zn treatment. The SUMOylated bands of ZIP8 are marked with arrows.

Given that the high Zn diet increased the mRNA and protein expression of ZIP8 and influenced SUMOylation modification in the intestine, we utilized database predictions to identify potential SUMOylation sites and mechanisms in ZIP8. We found that ZIP8 may serve as a target protein for SUMOylation modification by bioinformatic analysis. Therefore, immunoprecipitation was conducted to verify the SUMOylation status of ZIP8 and its binding affinity with SUMO1, SUMO2, and SUMO3 in yellow catfish. Immunoprecipitation analysis demonstrated that ZIP8 interacts with SUMO1, SUMO2, and SUMO3, suggesting that ZIP8 SUMOylation can be mediated by multiple SUMO isoforms (Figure 4A). Then, we employed Co-IP to verify whether the ZIP8 interacts with UBC9, which serves as the only E2 conjugating enzyme involved in the SUMOylation modification process [39]. The Co-IP analysis found the interaction between UBC9 and ZIP8, further validating that ZIP8 is capable of undergoing SUMOylation modification (Figure 4B,C).

#### 3.2.2. UBC9 Upregulated but SENP1 and Zn Downregulated the SUMOylation Level of ZIP8

Next, to investigate the role and mechanism of ZIP8 SUMOylation modification, the overexpression vectors of HA-ZIP8, His-UBC9, and Myc-SUMO1 were transfected into cells, and then the SUMOylation level of ZIP8 was detected by immunoprecipitation. The results showed that overexpression of UBC9 obviously upregulated the SUMOylation level of ZIP8, implying that UBC9 participate in SUMO1-mediated SUMOylation modification of ZIP8 (Figure 4D, Lane 4). Studies found that SUMOylation was a reversible process, with SENP1 mainly deSUMOylating SUMO1-mediated SUMOylation modification [40]. Thus, to verify its crucial role and mechanism of SENP1 in the deSUMOylation of ZIP8, HA-ZIP8, Myc-SUMO1, and His-UBC9 were co-transfected with Flag-SENP1 into HEK293T cells, and then the SUMOylation of ZIP8 was detected. In addition, our findings revealed that overexpressing SENP1 greatly decreased the SUMOylation level of ZIP8, suggesting that SENP1 participated in deSUMOylation modification of ZIP8 (Figure 4E, Lane 4).

Furthermore, to explore the effects of Zn incubation on the SUMOylation level of ZIP8, His-UBC9 and Myc-SUMO1 were co-transfected with HA-ZIP8 into HEK-293T cells and treated with or without 100 μM ZnSO_4_ for 24 h. The immunoprecipitation analysis showed that Zn incubation markedly decreased the SUMOylation modification of ZIP8 (Figure 4F, Lane 4). In summary, all the above results suggested that Zn addition significantly decreased the SUMOylation modification of ZIP8 protein.

#### 3.2.3. Lysine 24 (K24) and 222 (K222) Were the Major SUMOylation Modification Sites of ZIP8

**Figure 5:**
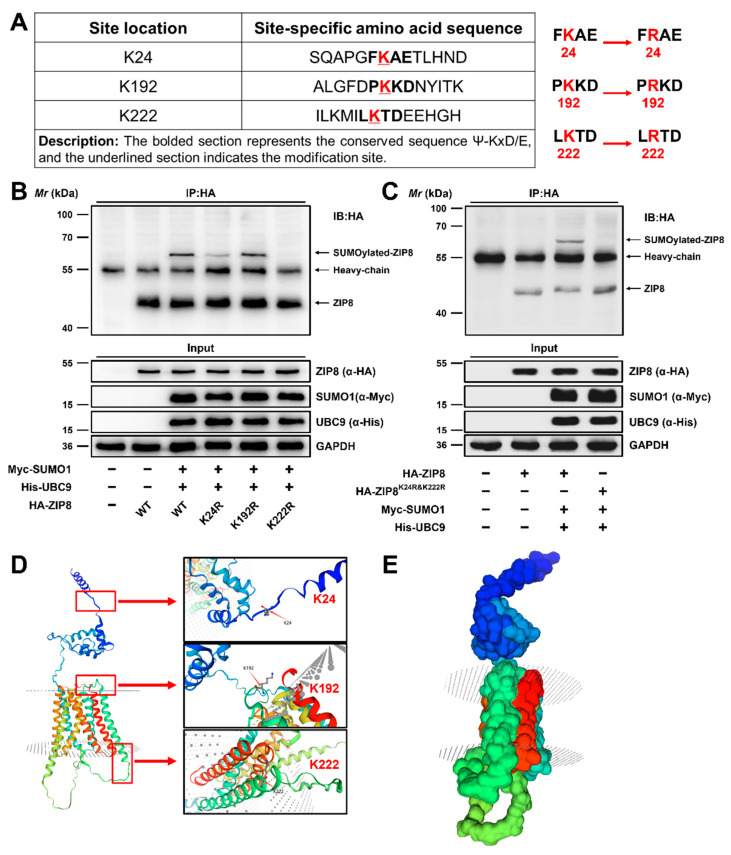
Lysine 24 (K24) and 222 (K222) are the major SUMOylation modification sites of ZIP8 in the yellow catfish. (**A**) SUMOylation modification sites and mutation information of ZIP8. (**B**,**C**) K24 and K222 were the major SUMOylation sites of ZIP8. (**D**,**E**) The predicted and constructed three-dimensional structure of ZIP8 and annotation of the SUMOylation modification sites.

The SUMOplot™ analysis program (http://www.abgent.com/sumoplot (accessed on 1 April 2023)) was utilized to forecast the potential SUMOylation modification sites of ZIP8 in yellow catfish, which were located on K24, K192, and K222 (Figure 5A). Previous studies have confirmed that the substitution of Lys (K) residue in the substrate protein with Arg (R) residue will block its binding to the SUMO molecule, thereby inhibiting the SUMOylation of the substrate protein [19,24]. To identify the most critical lysine residues for SUMOylation modification of ZIP8, we individually substituted the Lys (K) residue with Arg (R) residue (Figure 5A). Subsequently, overexpression plasmids of His-UBC9 and Myc-SUMO1 were co-transfected into HEK-293T cells along with the WT or mutant ZIP8 constructs, and the SUMOylation modification levels were analyzed as described above. Compared to the wild-type ZIP8, the SUMOylation level of ZIP8 remained largely unchanged in K192R mutant (Figure 5B, Lane 5), whereas the K24R and K222R mutants reduced the SUMOylation modification of ZIP8 (Figure 5B, Lanes 4 and 6). Subsequently, we further constructed a double-site deletion mutant of the ZIP8 protein, named K24R and K222R. Compared to the wild-type ZIP8, almost no SUMOylation modification was detected in the K24R and K222R groups (Figure 5C, Lane 4), confirming that the K24 and K222 positions were the main SUMOylation sites of ZIP8 in the yellow catfish. Moreover, we employed SWISS-MODEL homology modeling to predict the three-dimensional structure of ZIP8 in yellow catfish and annotated its three potential SUMOylation modification sites at K24, K192, and K222. Our findings revealed that ZIP8 possesses a transmembrane structure, with its main SUMOylation modification sites, K24 and K222, located on opposite sides of the membrane (Figure 5D,E).

#### 3.2.4. SENP1 Modulates the deSUMOylation Modification of ZIP8 by Sensing Zn-Induced Oxidative Stress

**Figure 6:**
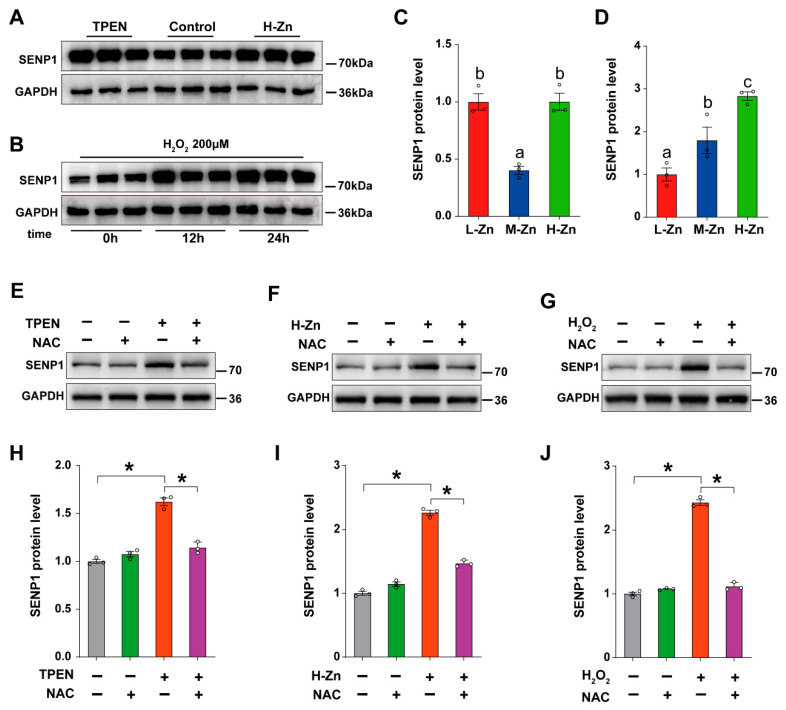
SENP1 regulates the SUMOylation level of ZIP8 by sensing oxidative stress. (**A**–**J**) Western blotting and quantitative analysis of changes in SENP1 under various treatments. Means ± SEM were reported from three independent biological experiments, with statistical significance assessed by Student’s *t*-test (* *p* < 0.05). Values with different letters (a–c) within the same chart are significantly different at *p* < 0.05.

Next, we explored the potential mechanistic link among Zn metabolism, oxidative stress, and SUMOylation modification. At first, the primary intestinal epithelial cells (IECs) isolated from yellow catfish were treated with 2 μm TPEN and 100 μM ZnSO_4_, respectively. In comparison to the control, TPEN and H-Zn groups greatly upregulated the protein expression of SENP1 (*p* < 0.05) (Figure 6A,C). Subsequently, IECs were exposed to H_2_O_2_ (200 μM) for 12 h and 24 h, and the expression of SENP1 protein was detected. Compared to the control, H_2_O_2_ treatment significantly upregulated the SENP1 protein expression at 12 h and 24 h (*p* < 0.05) (Figure 6B,D). All the above results indicate that Zn deficiency and excess, and oxidative stress, upregulated SENP1 expression.

Based on our in vivo results, we speculate that oxidative stress may mediate the response of SENP1 to Zn. To validate our hypothesis, the primary IECs were treated with an ROS Scavenger NAC (0.5 mM), Zinc chelator TPEN (2 μM), and ROS inducer H₂O₂ (200 μM). Compared with the control, the addition of TPEN upregulated the protein level of SENP1, while NAC co-incubation significantly alleviated the increase in SENP1 protein level induced by TPEN (*p* < 0.05), indicating that Zn deficiency induced the upregulation of SENP1 protein level by activating oxidative stress (Figure 6E,H). Compared with the control, H-Zn treatment elevated the protein abundance of SENP1, while pre-treatment with NAC markedly mitigated this H-Zn-induced upregulation in SENP1 protein levels (*p* < 0.05), suggesting that excessive Zn provokes upregulation of SENP1 protein expression via activation of oxidative stress (Figure 6F,I). Furthermore, NAC pre-treatment alleviated the increase in SENP1 protein level induced by H_2_O_2_-treatment (*p* < 0.05), indicating that oxidative stress promoted SENP1 protein expression (Figure 6G,J). Taken together, SENP1 can sense Zn deficiency- or excess-induced oxidative stress, increasing deSUMOylation modification of ZIP8, ultimately modulating Zn metabolism.

## 4. Discussion

Despite the established significance of SUMOylation in various biological processes, its potential involvement in the regulation of Zn metabolism remains mostly unexplored. Our current research discovered that high dietary Zn increases Zn concentration, affecting Zn metabolism and antioxidant capacity in the intestine. Furthermore, high Zn diet reduced SUMOylation modification by downregulating the expression of SUMO1, the E1 activating enzyme, Ubc9 conjugating enzyme, and E3 ligase, while concurrently enhancing the expression of SUMO-specific protease in the intestinal tissues and IECs of yellow catfish; SENP1 is capable of sensing Zn-induced oxidative stress and regulates the SUMOylation level of ZIP8. These observations not only indicate the crucial role of SENP1 in maintaining intestinal metabolic homeostasis and functional integrity, but also suggest a novel correlation between oxidative stress and Zn metabolism. Our study for the first time uncovered a regulatory pathway involving SUMOylation modification of ZIP8 through in vertebrates.

The intestinal Zn content was upregulated following increased dietary Zn intake, in agreement with previous studies [35,41]. Considering the impact of changes in Zn content on oxidative stress, the intestinal MDA content and antioxidant enzyme activity were analyzed [42]. Our investigation revealed that high dietary Zn induced intestinal oxidative stress, similar to other studies [14,35,43]. Zinc transporters are capable of responding to changes in external Zn concentration [44]. Our investigation revealed that elevated Zn levels in the diet upregulated the gene expression at the mRNA and protein levels for ZIP7, ZIP8, ZnT6, ZnT8, and ZnT1, indicating that high Zn increased intestinal Zn intake and affected intestinal Zn absorption and transport. Similarly, Yin et al. [11] indicated that elevated dietary Zn concentrations enhanced the gene expression related to Zn absorption and transport, which facilitated Zn transport and metabolism, and ultimately contributed to preserving the balance of Zn homeostasis.

Despite the established influence of Zn on the post-translational modification (PTM) of substrate proteins, research on the relationship between Zn and SUMOylation modification remains scarce [45]. SUMOylation is a crucial PTM process of target proteins, which was facilitated by SUMO protein and catalyzed by a series of key SUMOylating enzymes, thereby affecting the functions, localization, and interactions of target proteins [46]. Our study revealed that a high Zn diet reduced the SUMOylation activity by downregulating the expression of SUMO1 and UBC9 and upregulating that of SENP1, at both transcriptional and translational levels in the intestinal tissues, consistent with another study [34]. ZIP8 was mainly localized to the plasma membrane and is responsible for increasing the Zn content in the cytosol. Therefore, it is very important to explore whether and how ZIP8 undergoes SUMOylation modification, which can enhance our understanding of ZIP8’s role in regulating Zn homeostasis. For the SUMOylation of ZIP8, we demonstrated that SUMO1, SUMO2, and SUMO3 can each be conjugated to the protein. Similarly, Wu et al. [47] demonstrated that SUMO1, SUMO2, and SUMO3 each are conjugated to zinc finger homeobox 3 (ZFHX3). UBC9 is the only E2 conjugating enzyme for SUMOylation modification [39]. Our results indicated the interplay between UBC9 and ZIP8, further validating that ZIP8 is capable of undergoing SUMOylation modification in yellow catfish. In addition, our study found UBC9 upregulated the SUMOylation level of ZIP8 in yellow catfish. Similar studies revealed that the SUMOylation level of ZFHX3 depended on the UBC9 E2 conjugating enzyme [47]. The process of SUMOylation is reversible, and SENPs can mediate the removal of SUMOylation modification from the target proteins [24]. We found that SENP1 downregulated the SUMOylation level of ZIP8, suggesting that SENP1 participates in the deSUMOylation modification of ZIP8. Similarly, Wu et al. [47] reported that SENP1 has deSUMOylating activities for zinc finger homeobox 3 (ZFHX3). Zhao et al. [48] found that overexpression of SENP1 can reduce the SUMOylation of PKCε. Moreover, we found that Zn incubation reduced the SUMOylation level of ZIP8. Other studies have pointed out that SUMO1 contains Zn^2+^ binding sites and accordingly zinc can influence the stability of SUMO1 protein [49]. Furthermore, Zhang et al. [38] indicated that Zn regulated SUMOylation modification. Taken together, our findings suggested that Zn decreases the level of SUMOylation of ZIP8 through suppression of SUMO1 and enhancement of SENP1 expression.

SUMOylation, serving as a form of protein modification, involves the covalent attachment of a small ubiquitin-like protein to a lysine residue on target proteins [26]. Here, we innovatively found that the K24 and K222 positions are the primary SUMOylation modification sites of ZIP8 in the yellow catfish. In addition, we revealed that ZIP8 possesses a transmembrane structure, with its main SUMOylation modification sites, K24 and K222, located on opposite sides of the membrane. Thus, it is reasonable to speculate that the SUMOylation of ZIP8 at positions K24 and K222 was related to its subcellular localization. Studies have revealed that ZIP8 can reside on both the plasma membrane and the membranes of organelles [50]. Likewise, Hou et al. [51] indicated that SUMO1 facilitated the SUMOylation of YTHFD2 at two specific lysine residues: one positioned at the C-terminal (aa 384-579) and another at the N-terminal (aa 1-83). Hence, our study provided evidence that the SUMOylation modification may be implicated in modulating the subcellular localization of ZIP8.

Furthermore, we explored the link among Zn metabolism, oxidative stress, and SUMOylation modification. We found that Zn deficiency and excess, and H_2_O_2_ treatments, increased SENP1 protein expression, and this change could be reversed by the antioxidant NAC. This suggests that SENP1 can sense oxidative stress induced by Zn deficiency and excess, thereby enhancing the deSUMOylation level of ZIP8 and modulating its function in Zn absorption and transport. Similarly, NAC treatment substantially increased the SUMOylation in WT OT-I cells [52]. Moreover, the impact of H_2_O_2_ on SUMO conjugation is dose dependent. Previous studies found that most of the SUMO targets were dissociated at low doses (1 mM H_2_O_2_), while their binding remained unaffected at high concentrations and even showed a slight increase in the case of SUMO2 [53]. Other studies have found that SENP1 can participate in oxidative stress-related signaling pathways [24,40]. Dong et al. [54] found that hyperoxia promoted the expression of SENP1. Hence, we deeply conjectured that oxidative stress is involved in Zn-mediated upregulation of SENP1, which potentially regulates the subcellular localization of ZIP8. Hou et al. [51] revealed that SUMOylation of YTHDF2 at the major site of K571, both in vivo and in vitro, results in a reduction by oxidative stress. Furthermore, a recent study demonstrated that SENP1 enhanced the expression of antioxidant enzymes and concurrently diminished the generation of reactive oxygen species (ROS) [55]. Taken together, our research elucidated a novel function of SENP1 in mitigating Zn-induced oxidative damage, thereby suggesting that targeting SENP1 could represent a promising strategy for the modulation of Zn absorption and transport processes.

## 5. Conclusions

In conclusion, we discovered a new mechanism involving SENP1-mediated deSUMOylation of ZIP8 in the regulation of Zn homeostasis in intestine and intestinal epithelial cells. Our findings revealed that high Zn levels enhanced ZIP8 expression and induced intestinal Zn accumulation, ultimately triggering oxidative stress and activating SENP1. Furthermore, we confirmed that SUMO1 facilitates ZIP8 SUMOylation, while SENP1 catalyzes its deSUMOylation, with K24 and K222 serving as the primary SUMOylation sites on ZIP8. Furthermore, our results demonstrated that SUMO1 and SENP1 were the key enzymes facilitating the SUMOylation and deSUMOylation of ZIP8, respectively, with K42 and K222 serving as the main SUMOylation sites of ZIP8. Hence, targeting SENP1 to modulate the deSUMOylation of ZIP8 could potentially represent a novel mechanism regarding the regulation of Zn homeostasis.

## Data Availability

Data will be available from corresponding author upon reasonable request.

## Supplementary Materials

The following supporting information can be downloaded at: https://www.mdpi.com/article/10.3390/antiox14060750/s1, Text S1: Animal feeding, management, and sample collection; Text S2: Cell culture; Text S3: qRT-PCR; Text S4: Western blot analysis; Text S5: Immunoprecipitation and co-immunoprecipitation; Text S6: Plasmids construction and transfection; Table S1. Primers used for quantitative real-time PCR (qPCR) analysis; Table S2. Primers used for plasmid construction of expression vector.

## Abbreviations

CAT, Catalase; DMEM, Dulbecco’s Modified Eagle’s Medium; FBS, Fetal bovine serum; GAPDH, Glyceraldehyde-3-phosphate dehydrogenase; GPx, Glutathione peroxidase; HEK-293T, Human embryonic kidney 293T cell; ICP-OES, Inductively Coupled Plasma-Optical Emission Spectrometry; MDA, Malondialdehyde; MRE, Metal-responsive element; MT, Metallothionein; MTF-1, Metal-responsive transcription factor-1; PIAS1, E3 SUMO-protein ligase; qRT-PCR, Real-time quantitative PCR; SAE1, Sentrin/SUMO-specific Activating Enzyme Subunit 1; SAE2/UBA2, Sentrin/SUMO-specific Activating Enzyme Subunit 2; SEM, standard error of means; SENP1, Sentrin/SUMO-specific protease 1; SUMO, Sentrin/small ubiquitin-related modifier; T-AOC, Total antioxidant capacity; TPEN, N,N,N′,N′-Tetrakis (2-pyridylmethyl)ethylenediamine; UBC9, Ubiquitin-Conjugating Enzyme 9; ZIP, Zinc-regulated transporters, iron-regulated transporter-like protein; Zn, Zinc; ZnT, Solute carrier family 30 (zinc transporter).

## References

[1] Ho E., Wong C.P., King J.C. (2022). Impact of zinc on DNA integrity and age-related inflammation. Free Radic. Biol. Med..

[2] Zackular J.P., Moore J.L., Jordan A.T., Juttukonda L.J., Noto M.J., Nicholson M.R., Crews J.D., Semler M.W., Zhang Y., Ware L.B. (2016). Dietary zinc alters the microbiota and decreases resistance to clostridium difficile infection. Nat. Med..

[3] Duan M., Li T., Liu B., Yin S., Zang J., Lv C., Zhao G., Zhang T. (2023). Zinc nutrition and dietary zinc supplements. Crit. Rev. Food Sci. Nutr..

[4] Wiuf A., Steffen J.H., Becares E.R., Grønberg C., Mahato D.R., Rasmussen S.G.F., Andersson M., Croll T., Gotfryd K., Gourdon P. (2022). The two-domain elevator-type mechanism of zinc-transporting ZIP proteins. Sci. Adv..

[5] Kambe T., Tsuji T., Hashimoto A., Itsumura N. (2015). The physiological, biochemical, and molecular roles of zinc transporters in zinc homeostasis and metabolism. Physiol. Rev..

[6] Yu F., Hou Z.S., Luo H.R., Cui X.F., Xiao J., Kim Y.B., Li J.L., Feng W.R., Tang Y.K., Li H.X. (2022). Zinc alters behavioral phenotypes, neurotransmitter signatures, and immune homeostasis in male zebrafish (*Danio rerio*). Sci. Total Environ..

[7] Maares M., Haase H.A. (2020). Guide to human zinc absorption: General overview and recent advances of in vitro intestinal models. Nutrients.

[8] Pei X., Jiang H., Li C., Li D., Tang S. (2023). Oxidative stress-related canonical pyroptosis pathway, as a target of liver toxicity triggered by zinc oxide nanoparticles. J. Hazard. Mater..

[9] Gavino F., Valeria M.N., Alberto R., Daniela F., Sonia N., Clara G., Van Eyken P., Geboes K. (2008). Zinc in gastrointestinal and liver disease. Coord. Chem. Rev..

[10] Bafaro E., Liu Y., Xu Y., Dempski R.E. (2017). The emerging role of zinc transporters in cellular homeostasis and cancer. Signal Transduct. Target. Ther..

[11] Yin S., Duan M., Fang B., Zhao G., Leng X., Zhang T. (2023). Zinc homeostasis and regulation: Zinc transmembrane transport through transporters. Crit. Rev. Food. Sci. Nutr..

[12] Hernández-Camacho J.D., Vicente-García C., Parsons D.S., Navas-Enamorado I. (2020). Zinc at the crossroads of exercise and proteostasis. Redox Biol..

[13] Krezel A., Maret W. (2007). Dual nanomolar and picomolar Zn(II) binding properties of metallothionein. J. Am. Chem. Soc..

[14] Hübner C., Haase H. (2021). Interactions of zinc- and redox-signaling pathways. Redox Biol..

[15] Sims H.I., Chirn G.W., Marr M.T. (2012). 2nd. Single nucleotide in the MTF-1 binding site can determine metal-specific transcription activation. Proc. Natl. Acad. Sci. USA.

[16] He L., Girijashanker K., Dalton T.P., Reed J., Li H., Soleimani M., Nebert D.W. (2006). ZIP8, member of the solute-carrier-39 (SLC39) metal-transporter family: Characterization of transporter properties. Mol. Pharmacol..

[17] Liu M.J., Bao S., Galvez-Peralta M., Pyle C.J., Rudawsky A.C., Pavlovicz R.E., Killilea D.W., Li C., Nebert D.W., Wewers M.D. (2013). ZIP8 regulates host defense through zinc-mediated inhibition of NF-κB. Cell Rep..

[18] Chen C.H., Chang C.C., Lee T.H., Luo M., Huang P., Liao P.H., Wei S., Li F.A., Chen R.H., Zhou X.Z. (2013). SENP1 deSUMOylates and regulates Pin1 protein activity and cellular function. Cancer Res..

[19] Barry R., John S.W., Liccardi G., Tenev T., Jaco I., Chen C.H., Choi J., Kasperkiewicz P., Fernandes-Alnemri T., Alnemri E. (2018). SUMO-mediated regulation of NLRP3 modulates inflammasome activity. Nat. Commun..

[20] Huang C.H., Yang T.T., Lin K.I. (2024). Mechanisms and functions of SUMOylation in health and disease: A review focusing on immune cells. J. Biomed. Sci..

[21] Jackson S.P., Durocher D. (2013). Regulation of DNA damage responses by ubiquitin and SUMO. Mol. Cell.

[22] Kunadis E., Lakiotaki E., Korkolopoulou P., Piperi C. (2021). Targeting post-translational histone modifying enzymes in glioblastoma. Pharmacol. Ther..

[23] Mukhopadhyay D., Dasso M. (2007). Modification in reverse: The SUMO proteases. Trends Biochem. Sci..

[24] Wang T., Cao Y., Zheng Q., Tu J., Zhou W., He J., Zhong J., Chen Y., Wang J., Cai R. (2019). SENP1-Sirt3 signaling controls mitochondrial protein acetylation and metabolism. Mol. Cell.

[25] Du F.L., Dong W.B., Zhang C., Li Q.P., Kang L., Lei X.P., Guo L., Zhai X.S. (2019). Budesonide and poractant alfa prevent brochopulmonary dysplasia via triggering SIRT1 signaling pathway. Eur. Rev. Med. Pharmacol. Sci..

[26] Yang Y., Fu W., Chen J., Olashaw N., Zhang X., Nicosia S.V., Bhalla K., Bai W. (2007). SIRT1 sumoylation regulates its deacetylase activity and cellular response to genotoxic stress. Nat. Cell. Biol..

[27] Su G., Logez M., Xu J., Tao S., Villéger S., Brosse S. (2021). Human impacts on global freshwater fish biodiversity. Science.

[28] Hotamisligil G.S. (2006). Inflammation and metabolic disorders. Nature.

[29] Chen G.H., Song C.C., Zhao T., Hogstrand C., Wei X.L., Lv W.H., Song Y.F., Luo Z. (2022). Mitochondria-dependent oxidative stress mediates ZnO nanoparticle (ZnO NP)-induced mitophagy and lipotoxicity in freshwater teleost fish. Environ. Sci. Technol..

[30] Gong G., Dan C., Xiao S., Guo W., Huang P., Xiong Y., Wu J., He Y., Zhang J., Li X. (2018). Chromosomal-level assembly of yellow catfish genome using third-generation DNA sequencing and Hi-C analysis. GigaScience.

[31] Wei X., Hogstrand C., Chen G., Lv W., Song Y., Xu Y., Luo Z. (2021). Zn induces lipophagy via the deacetylation of beclin1 and alleviates Cu-induced lipotoxicity at their environmentally relevant concentrations. Environ. Sci. Technol..

[32] Song C.C., Wu L.X., Chen G.H., Lv W.H., Chen S.W., Luo Z. (2020). Six members of SLC30A/ZnTs family related with the control of zinc homeostasis: Characterization, mRNA expression and their responses to dietary ZnO nanoparticles in yellow catfish. Aquaculture.

[33] Liu Y.C., Lin M.C., Chen H.C., Tam M.F., Lin L.Y. (2011). The role of small ubiquitin-like modifier-interacting motif in the assembly and regulation of metal-responsive transcription factor 1. J. Biol. Chem..

[34] Song C.C., Liu T., Hogstrand C., Zhong C.C., Zheng H., Sun L.H., Luo Z. (2024). SENP1 mediates zinc-induced ZnT deSUMOylation at Lys-409 involved in the regulation of zinc metabolism in Golgi apparatus. Cell. Mol. Life Sci..

[35] Luo Z., Tan X.Y., Zheng J.L., Chen Q.L., Liu C.X. (2011). Quantitative dietary zinc requirement of juvenile yellow catfish *Pelteobagrus fulvidraco*, and effects on hepatic intermediary metabolism and antioxidant responses. Aquaculture.

[36] Zhao T., Yang S.B., Chen G.H., Xu Y.H., Xu Y.C., Luo Z. (2020). Dietary glucose increases glucose absorption and lipid deposition via SGLT1/2 signaling and acetylated ChREBP in the intestine and isolated intestinal epithelial cells of yellow catfish. J. Nutr..

[37] Xu Y.C., Pantopoulos K., Zheng H., Zito E., Zhao T., Tan X.Y., Wei X.L., Song Y.F., Luo Z. (2023). Phosphorus overload promotes hepatic lipolysis by suppressing GSK3β-dependent phosphorylation of PPARα at Ser84 and Thr265 in a freshwater teleost. Environ. Sci. Technol..

[38] Zhang X., Wang C., Zhao D., Chen X., Zhang C., Zheng J., Liu X. (2020). Zinc deficiency induces abnormal development of the myocardium by promoting SENP5 overexpression. PLoS ONE.

[39] Bernier-Villamor V., Sampson D.A., Matunis M.J., Lima C.D. (2002). Structural basis for E2-mediated SUMO conjugation revealed by a complex between ubiquitin-conjugating enzyme Ubc9 and RanGAP1. Cell.

[40] Jiao Y., Zhang X., Yang Z. (2024). SUMO-specific proteases: SENPs in oxidative stress-related signaling and diseases. Biofactors.

[41] Xu Y.C., Zheng H., Guo J.C., Tan X.Y., Zhao T., Song Y.F., Wei X.L., Luo Z. (2023). Effects of different dietary Zinc (Zn) sources on growth performance, Zn metabolism, and intestinal health of grass Carp. Antioxidants.

[42] Marreiro D.D., Cruz K.J., Morais J.B., Beserra J.B., Severo J.S., de Oliveira A.R. (2017). Zinc and oxidative stress: Current mechanisms. Antioxidants.

[43] Meiler K.A., Cleveland B., Radler L., Kumar V. (2021). Oxidative stress-related gene expression in diploid and triploid rainbow trout (*Oncorhynchus mykiss*) fed diets with organic and inorganic zinc. Aquaculture.

[44] Chen S.W., Wu K., Lv W.H., Song C.C., Luo Z. (2020). Molecular characterization of ten zinc (Zn) transporter genes and their regulation to Zn metabolism in freshwater teleost yellow catfish *Pelteobagrus fulvidraco*. J. Trace Elem. Med. Biol..

[45] Xu Y.C., Zheng H., Hogstrand C., Tan X.Y., Zhao T., Song Y.F., Wei X.L., Wu L.X., Luo Z. (2023). Novel mechanism for zinc inducing hepatic lipolysis via the HDAC3-mediated deacetylation of β-catenin at lysine 311. J. Nutr. Biochem..

[46] Melchior F., Schergaut M., Pichler A. (2003). SUMO: Ligases, isopeptidases and nuclear pores. Trends Biochem. Sci..

[47] Wu R., Fang J., Liu M., A J., Liu J., Chen W., Li J., Ma G., Zhang Z., Zhang B. (2020). SUMOylation of the transcription factor ZFHX_3_ at Lys-2806 requires SAE_1_, UBC_9_, and PIAS_2_ and enhances its stability and function in cell proliferation. J. Biol. Chem..

[48] Zhao X., Xia B., Cheng J., Zhu M.X., Li Y. (2020). PKCε SUMOylation is required for mediating the nociceptive signaling of inflammatory pain. Cell Rep..

[49] Kaur A., Jaiswal N., Raj R., Kumar B., Kapur S., Kumar D., Gahlay G.K., Mithu V.S. (2020). Characterization of Cu^2+^ and Zn^2+^ binding sites in SUMO1 and its impact on protein stability. Int. J. Biol. Macromol..

[50] Wang J., Zhao H., Xu Z., Cheng X. (2020). Zinc dysregulation in cancers and its potential as a therapeutic target. Cancer Biol. Med..

[51] Hou G., Zhao X., Li L., Yang Q., Liu X., Huang C., Lu R., Chen R., Wang Y., Jiang B. (2021). SUMOylation of YTHDF2 promotes mRNA degradation and cancer progression by increasing its binding affinity with m^6^A-modified mRNAs. Nucleic Acids Res..

[52] Wu Z., Huang H., Han Q., Hu Z., Teng X.L., Ding R., Ye Y., Yu X., Zhao R., Wang Z. (2022). SENP7 senses oxidative stress to sustain metabolic fitness and antitumor functions of CD8^+^ T cells. J. Clin. Investig..

[53] Bossis G., Melchior F. (2006). Regulation of SUMOylation by reversible oxidation of SUMO conjugating enzymes. Mol. Cell.

[54] Dong W., Zhu X., Liu X., Zhao X., Lei X., Kang L., Liu L. (2021). Role of the SENP1-SIRT1 pathway in hyperoxia-induced alveolar epithelial cell injury. Free Radic. Biol. Med..

[55] Xia K., Guo J., Yu B., Wang T., Qiu Q., Chen Q., Qiu T., Zhou J., Zheng S. (2024). Sentrin-specific protease 1 maintains mitochondrial homeostasis through targeting the deSUMOylation of sirtuin-3 to alleviate oxidative damage induced by hepatic ischemia/reperfusion. Free Radic. Biol. Med..

