# Supplementary material for: SENP1 Senses Oxidative Stress to Regulate the SUMOylation Modification of ZIP8 and Maintain Zinc Transport Functions
Source: Antioxidants (Basel). 2025 Jun 18;14(6):750. doi: 10.3390/antiox14060750 (PMC12190111; doi:10.3390/antiox14060750)
Supplement: Supplementary file 1 [file antioxidants-14-00750-s001.zip › antioxidants-3617920-supplementary.pdf]

SENP1 senses oxidative stress to regulate the SUMOylation modification of ZIP8 and maintain zinc transport functions

Tao Liu [1, a], Chang-Chun Song [1, a], Fu-Xuan Duan [1], Chong-Chao Zhong [1], Sheng-Zan Liu [1], Jia-Cheng Guo [1], An-Gen Yu [1], Zhi Luo [1,2, *]

[1]Hubei Hongshan Laboratory, Fishery College, Huazhong Agriculture University, Wuhan 430070, China

[2]Laboratory for Marine Fisheries Science and Food Production Processes, Qingdao Marine Science and Technology Center, Qingdao 266237, China

[*] Corresponding author: Prof. Zhi Luo, Tel.: +86-27-8728-2113; Fax: +86-27-8728-2114; Email address: luozhi99@mail.hzau.edu.cn; luozhi99@aliyun.com (Z. Luo).

[a] These two authors contributed equally to this work.

Text S1

Animal feeding, management, and sample collection

Uniformly-sized juvenile yellow catfish (initial mean weight: 4.97 ± 0.01 g, mean ± SEM, one-month old) were randomly distributed into nine circular tanks (each with a water volume of 300 L), with 30 fish per tank, after a two-week acclimation period. Each diet was assigned to triplicate tanks, and the fish were fed to satiation twice daily (8:30 and 16:30). Water quality was monitored twice weekly during the experiment. The experiment lasted for 10 weeks.

At the end of the 10-week feeding experiment, all experimental fish were fasted for 24 hours before sampling to avoid the effects of feeding. Then, the yellow catfish were euthanized using 100 mg/L MS-222 (#E10521, Sigma-Aldrich, St. Louis, MO, USA). Fish from each tank were retrieved, weighed, and dissected on ice to obtain intestinal tissues. Nine yellow catfish were randomly collected from each tank, and the intestinal tissues were quickly frozen in liquid nitrogen and stored in a -80°C freezer for subsequent analysis of intestinal zinc content, enzyme activity, mRNA, and protein

expression.

Text S2

Cell culture

HEK293T cell lines were obtained from the Cell Resource Center of Huazhong Agricultural University. The primary intestinal epithelial cells (IECs) and HEK-293T cells were grown in Dulbecco's Modified Eagle Medium (DMEM, #11965092, Thermo Fisher Scientific) supplemented with 10% fetal bovine serum (FBS, #10099141, Thermo Fisher Scientific), within a humidified incubator maintained at 37°C with 5% CO_2.

Text S3

qRT-PCR

Agarose gel electrophoresis was employed to verify the integrity of the total RNA samples. The purity of the total RNA was measured using a Nanodrop ND-2000 spectrophotometer (Thermo Fisher Scientific, Waltham, MA, USA) to determine the values of OD_{260}, OD_{280}, and OD_{230} ($OD_{260}/OD_{280} \geq 1.8$, $OD_{260}/OD_{230} \geq 1.5$). Using a Reverse Transcription Kit (TaKaRa, Tokyo, Japan), 1 microgram of total RNA was transcribed into cDNA in a 25-microliter system, consisting of 12.5 microliters of qPCR Mix (#RR430, TaKaRa), 0.5 microliters of each forward and reverse primer (10 mM), 1 microliter of diluted cDNA template, and 10.5 microliters of double-distilled H_2O. The PCR parameters included an initial denaturation step at 95°C for 30 seconds, followed by 45 cycles of 5 seconds at 95°C and 15 seconds at 60°C. After quantitatively analyzing the mRNA expression of 18S ribosomal RNA (18S rRNA), beta-2-microglobulin (b2m), elongation factor 1α (elf1α), glyceraldehyde-3-phosphate dehydrogenase (gapdh), hypoxanthine phosphoribosyl transferase (hprt), ribosomal protein L7 (rpl7), TATA-box binding protein (tbp), tubulin alpha chain (tuba), ubiquitin-conjugating enzyme (ubce), beta-actin (β-actin), and the most stable two genes as the controls were selected via geNorm software (https://genorm. cmgg. be/).

Text S4

Western blot analysis

In Western blot analysis, intestinal and cell samples were lysed in RIPA buffer

(#P0013B, Beyotime Biotechnology, Shanghai, China) with PMSF protease inhibitor (#P1045, Beyotime Biotechnology). The protein concentration of the supernatant was determined using a BCA protein assay kit (#23225, Thermo Fisher Scientific). Protein samples (20 μg) were loaded onto SDS-polyacrylamide gels and then transferred to polyvinylidene fluoride (PVDF) membranes. The membranes were blocked with TBST solution containing 8% skim milk powder for 1.5 hours at room temperature and then incubated with primary antibodies overnight at 4°C. The next day, the membranes were incubated with the corresponding secondary antibodies for 1 hour at room temperature. After further washing, the membranes were visualized using ECL (#P0018FS, Beyotime Biotechnology, Shanghai, China). These antibodies include anti-GAPDH (1:10000, #10494-1-AP, Proteintech, Wuhan, China), anti-ZIP7 (1:1000, #19429-1-AP, Proteintech, Wuhan, China), anti-ZIP8 (1:2000, #20459-1-AP, Proteintech, Wuhan, China), anti-ZnT1 (1:2000, #A9954, ABclonal, Wuhan, China), anti-ZnT6 (1:2000, #13526-1-AP, Proteintech, Wuhan, China), anti-ZnT8 (1:1000, #16169-1-AP, Proteintech, Wuhan, China), anti-SUMO1 (1:1000, #10329-1-AP, Proteintech, Wuhan, China), anti-SUMO2/3 (1:1000, # 11251-1-AP, Proteintech, Wuhan, China), anti-SAE1 (1:1000, #10229-1-AP, Proteintech, Wuhan, China), anti-UBC9 (1:2000, #10070-1-AP, Proteintech, Wuhan, China), anti-PIAS1 (1:2000, #23395-1-AP, Proteintech, Wuhan, China), anti-SENP1 (1:2000, #25349-1-AP, Proteintech, Wuhan, China), anti-SENP2 (1:1000, # A17994, ABclonal, Wuhan, China), anti-Myc-tag (1:3000, #AE070, ABclonal, Wuhan, China), anti-6 × His-tag (1:5000, #66005-1-lg, Proteintech, Wuhan, China), anti-HA-tag (1:5000, #GS20004, Mabnus, Wuhan, China), anti-Flag-tag (1:5000, #80010-1-RR, Proteintech, Wuhan, China), HRP-conjugated Goat anti-Rabbit IgG (H+L) (1:10000, #7074, Cell Signaling Technology), and HRP-conjugated Goat anti-Mouse IgG (H+L) (1:10000, #AS003, ABclonal, Wuhan, China). All experiments were repeated at least three times to ensure the reliability of the results.

Text S5

Immunoprecipitation and co-immunoprecipitation

To conduct immunoprecipitation and co-immunoprecipitation analyses, after different treatments, the cells were washed twice with cold PBS and then lysed in NP-

40 lysis buffer (#P0013F, Beyotime Biotechnology, Shanghai, China) supplemented with 1% protease inhibitor cocktail (#P1005, Beyotime Biotechnology, Shanghai, China). To detect SUMOylated proteins, N-ethylmaleimide (NEM, #E3876, 20 mM, Sigma, St Louis, MO, USA) was added to the lysis buffer to preserve SUMOylation of ZIP8 during the experiment. After centrifugation at 12000 g for 15 minutes, the cell lysates were incubated with corresponding antibodies overnight at 4°C. Protein A/G magnetic beads (#HY-K0202, Med Chem Express, New Jersey, USA) were then added and incubated for 4 hours at 4°C. After washing with cold lysis buffer five times, the immune complexes were collected by centrifugation at 2000 rpm for 2 minutes, boiled in 1×SDS loading buffer for 5-10 minutes, and then subjected to western blotting analysis.

Text S6

Plasmids construction and transfection

In brief, we utilized the ClonExpress II One Step Cloning Kit (#C112, Vazyme, Nanjing, China) to subclone the open reading frames of ZIP8, SUMO1/2/3, UBC9, and SENP1 into pcDNA3.1 vectors with HA-tag, Myc-tag, His-tag, and Flag-tag sequences, respectively, following the manufacturer's instructions. Mutations of lysine (K) residues K24, K192, and K222 in the full-length pcDNA3.1-HA-ZIP8 plasmid were generated into arginine (R) residues using the Mut Express II Fast Mutagenesis Kit (#C214, Vazyme, Nanjing, China) and confirmed by DNA sequencing. Subsequently, the plasmids were transfected into HEK293T cells using Lipo293™ transfection reagent (#C0521, Beyotime Biotechnology, Shanghai, China) according to the manufacturer's instructions.

Table S1. Primers used for quantitative real-time PCR (qPCR) analysis.

Gene	Forward primer (5'-3')	Reverse primer (5'-3')	NCBI number
zip1	TCATTGTTTTCCACTGT GGTT	ACACACCTGCAGAGAT ACAGC	XM_027154968.1
zip3	GACGGTGCTAAATTAGG AAGC	CTTGGAGCACCACAGA AATAA	XM_027169847.1
zip4	CAGATGTACCAGAAGG AGCAA	CTGCGAAGTTATGAATG CTGT	XM_027175519.1
zip5	GGACTTTACCTGCTCTT CACA	GACTCAGGGTTCATAGA GCAA	XM_027134554.1
zip6	TGGAAAAAGCCAGAAG AATTT	CCTGAGAATGTGGGATA AGGT	XM_027137915.1
zip7	GACTCAGTGGAGTTGT GGATG	AGCATGAGGTATGAGAT GCAG	XM_027139213.1
zip8	TTATTGGAGACCGTCAC TCAG	AGCCATAACCCCATACT TGAC	XM_027138204.1
zip9	GCACTGTATGAGGAGG TGCTA	CATGAAGACAAATCCC AACAC	XM_027173187.1
zip10	GCTCATGTAGTCCGAAT CTCA	TCATAAACAACGCTTCG AGTC	XM_027152464.1
zip11	ATGGAGATGTTCACCAC AGAA	CCTCAGGAATGTTGTGT ATGG	XM_027159100.1
zip12	CTCTAACCTCGTGGAAC AAAA	TGTCCTCCACTGGTTGT ATTT	XM_027169508.1
zip13	CCAGCAACAATTCAAG AGAGA	TGATCTGCTGAAGAGG AAATG	XM_027137129.1
zip14	AACGACTGCTGCTCTAC TTCA	GTTTCCCACAGGAAAA TGACT	XM_027162824.1
znt1	ACATTCACTGCAATGAC CAAT	TCGTGTACAGAACAATG CAGA	XM_027166182.1
znt2	GCTATCACAAGATCACC GAGA	AGAGAACAGGAAGGTG CAGAT	XM_027137956.1
znt4	GCTGTCTGCAGGTATCA GTGT	GGTTGAGGAGAAATCC CATTA	XM_027158877.1
znt5	TTCTCTTTACCACCTCA GACG	ACAACTCCACCTTTATG GTCA	XM_027143665.1
znt6	GGATGAATCCCTTTGTC CTTA	AAGGAGTGGTCTGGAG AAGAA	XM_027174530.1
znt7	TGACAAGACATCACGG AATTT	ACGAAACCTGCCAGTA CTTCT	XM_027159169.1
znt8	AACCATCGTCAAAGTG TGGTA	TAAAGGCGTCGACAAT AACTG	MK844296

znt9	CATCTCACGCATATGCTTCTT	CTTAGGCTCTTCGCTCTGATT	XM_027135373.1
znt10	ATCAGGAACGTCCTCCATAAC	TCTCGGTGCTTGTATTTTGAG	XM_027165809.1
mt	ATCCTTGCGAGTGCTCCA	GCAGGAATCGCCCTTACAC	EU124661.1
mtf-1	TGTGCCTCAGTGATTTGAGC	CCTCCACCAGGTTGTCTGAT	XM_027148917.2
sumo1	TGACAACGCATTTAAAGAAGC	CTGATACACCTCGATCACGTC	XM_027160137.1
sumo2	CTGATGAAGGCCTACTGTGAA	TCAAATCACACCTCCTGTCTG	XM_027171226.1
sumo3	CGCTCAGCAAGTTAATGAAAG	GCCCGTTTGTTGTTGAAATAC	XM_027159896.1
sae1	TCAAGTAGAAAGCAAGCCAGA	TATCCATGGTAACCGAAGACA	XM_027147771.1
sae2	AGCGCATTTCCACTAAAGACT	GTCTTGTTTGTTCACGAGCTG	XM_027167905.2
ubc9	GTTTTGTAGCTGTGCCGACTA	ATTTACATTTGGGAGGAGACG	MH192980.1
pias1	ACCAAATGTGACTTCACGGTA	CCAGTGAGGTAATGTTGATGG	MH192981.1
senp1	TGACAAGCCTTTATGATGCTC	GAATTCCTGTTCCTCTGCTTC	XM_027144549.1
senp2	GTCGACCTCTTCCTCTACGAC	GGTCACCGTCCACTTTAAGAT	XM_027160022.1
senp3	TTAACTACAAGCGCCATGTTC	AATGGGAATCAACAGAAGGTC	MH192984.1
senp5	GGCTGGAAGATGACGGTCAA	AGGTCAGTGAGCTTGCAGTC	XM_027169964.2
senp6	TAGATCAGAAGCCAGCAGAGA	GTTCCCAGACTTGAAATGGTT	XM_027177148.1
senp7	AGGTCTCCGACATGGAAATAC	AAGGTGTAGACGCTGGAGTTT	XM_027143618.1
senp8	TGAGTTATCAGGACAGCTTGC	AGCCTCAGTGGCTCTAAGAAG	XM_027153181.1
18s rRNA	AGCTCGTAGTTGGATCTCGG	CGGGTATTCAGGCGAGTTTG	KP938527
b2m	GCTGATCTGCCATGTGAGTG	TGTCTGACACTGCAGCTGTA	KP938520
elfa	GTCTGGAGATGCTGCCATTG	AGCCTTCTTCTCAACGCTCT	KU886307
gapdh	TTTCAGCGAGAGAGACCCAG	ATGACTCTCTTGGCACCTCC	KP938521

hprt	ATGCTTCTGACCTGGAA CGT	TTGCGGTTCAGTGCTTT GAT	KP938523
rpl7	GGCAAATGTACAGGAG CGAG	GCCTTGTTGAGCTTGAC GAA	KP938522
tbp	AGCAAAGAGTGAGGAG CAGT	ACTGCTGATGGGTGAG AACA	KP938525
tuba	TCAAAGCTGGAGTTCT CGGT	AATGGCCTCGTTATCCA CCA	KP938526.1
ubce	TCAAGAAGAGCCAGTG GAGG	TAGGGGTAGTCGATGG GGAA	KP938524
β-actin	GGACTCTGGTGATGGT GTGA	CTGTAGCCTCTCTCGGT CAG	EU161066

Abbreviations: ***18S rRNA***, 18S ribosomal RNA; ***b2m***, beta-2-microglobulin; ***elfa***, elongation factor 1a; ***gapdh***, glyceraldehyde-3-phosphate dehydrogenase; ***hprt***, hypoxanthine phosphoribosyl transferase; ***mt***, metallothionein; ***mtf-1***, metal regulatory transcription factor 1; ***pias1***, E3 SUMO-protein ligase; ***rpl7***, ribosomal protein L7; ***sae1***, Sentrin/SUMO-specific activating enzyme subunit 1; ***sae2***, Sentrin/SUMO-specific activating enzyme subunit; ***senp***, Sentrin/SUMO-specific protease; ***sumo***, Sentrin/small ubiquitin-related modifier; ***tbp***, TATA-box binding protein; ***tuba***, tubulin alpha chain; ***ubc9***, Ubiquitin-Conjugating Enzyme 9; ***ubce***, ubiquitin-conjugating enzyme; ***zip1, 3, 4, 5, 6, 7, 8, 9, 10, 11, 12, 13, 14***, solute carrier family 39 member1, 3, 4, 5, 6, 7, 8, 9, 10, 11, 12, 13, 14; ***znt1, 2, 4, 5, 6, 7, 8, 9, 10***, solute carrier family 30 member1, 2, 4, 5, 6, 7, 8, 9, 10; ***β-actin***, beta-actin.

Table S2. Primers used for plasmid construction of expression vector.

	Forward primer (5'-3')	Reverse primer (5'-3')
pcDNA3.1-ZIP8-HA-1	GTTCACTGCGACAGAAGACAC	TCAAGCGTAATCTGGAACATCGTATGGGTAGCCCAGGTTAAT
pcDNA3.1-ZIP8-HA-2	ctagcgtttaaacttaagcttATGAACGACTTTATCATCCTCTGTGC	aacgggccctctagactcgagTCAAGCGTAATCTGGAACATCG
pcDNA3.1-SUMO1-Myc-1	TCTAATCTCCTCCTTACTCCA	CTACAGATCCTCTTCAGAGATGAGTTTCTGCTCGTCGTTCCAGT
pcDNA3.1-SUMO1-Myc-2	ctagcgtttaaacttaagcttATGTCAGACACGGAGACAAAACC	aacgggccctctagactcgagCTACAGATCCTCTTCAGAGATGAGTTTC
pcDNA3.1-SUMO2-Myc-1	TTCAGCCAATCGTAGAGCA	TCACAGATCCTCTTCAGAGATGAGTTTCTGCTCAATCACACCTCC
pcDNA3.1-SUMO2-Myc-2	ctagcgtttaaacttaagcttATGGCGGACGAGAAGCCC	aacgggccctctagactcgagTCACAGATCCTCTTCAGAGATGAGTT
pcDNA3.1-SUMO3-Myc-1	TTAACAGCGCATCTTGTGTAA	CTACAGATCCTCTTCAGAGATGAGTTTCTGCTCGCGATGGCCGCCCGTT
pcDNA3.1-SUMO3-Myc-2	ctagcgtttaaacttaagcttATGTCTGAGGATAAGCCCAAGG	aacgggccctctagactcgagCTACAGATCCTCTTCAGAGATGAGTTTC
pcDNA3.1-UBC9-His-1	ACTGTTTGGAGTTTCATTCACC	TTAATGGTGATGGTGATGATGGGGAGAAAACTTTTTGGCTTG
pcDNA3.1-UBC9-His-2	ctagcgtttaaacttaagcttATGTCTGGTATAGCGCTGAGTCG	aacgggccctctagactcgagTTAATGGTGATGGTGATGATGGG
pcDNA3.1-SENP1-Flag-1	CGTGCGTTACTTATCGGTG	TTACTTATCGTCGTCATCCTTGTAATCTCGTAGAAGTTTCCGGTT
pcDNA3.1-SENP1-Flag-2	ctagcgtttaaacttaagcttATGAGCTTCAACATGTTCAATAAATTC	aacgggccctctagactcgagTTACTTATCGTCGTCATCCTTGTAATC
ZIP8-K24R mutation	GGTTTcgaGCTGAGACTTTACACAATGATTTCCT	AGTCTCAGCtcgAAACCCGGGAGCTTGACTTAG
ZIP8-K192R mutation	TGACCCGcgaAAAGATAACTACATCACGAAGGCTGT	TATCTTTtcgCGGGTCAAACCCCAGAGCCTCA
ZIP8-K222R mutation	TGATCCTAcgaACCGACGAAGAGCACGGCCAC	GTCGGTtcgTAGGATCATCTTCAGGATCCTCTCC
